# A Rare Case of Mass-Like Hypertrophic Cardiomyopathy

**DOI:** 10.7759/cureus.12787

**Published:** 2021-01-19

**Authors:** Juwairiya Shuroog, Justin Canakis, Fawad J Khan, Prakash Suryanarayana, Shahabuddin Soherwardi

**Affiliations:** 1 Internal Medicine, TidalHealth, Salisbury, USA; 2 Internal Medicine, Philadelphia College of Osteopathic Medicine, Philadelphia, USA; 3 Department of Internal Medicine, Howard University Hospital, Washington DC, USA

**Keywords:** hypertrophic cardiomyopathy, mass, fibroma, hcm, hocm

## Abstract

Mass-like hypertrophic cardiomyopathy (HCM) is a unique variant of HCM. HCM predominantly causes mid-ventricular, concentric hypertrophy, and asymmetric septal hypertrophy; however, focal hypertrophy mimicking a cardiac fibroma is rare. A 29-year-old female with a past medical history of recurrent orthostatic hypotension and syncope presented to the emergency department (ED) complaining of lightheadedness, dizziness, and generalized weakness associated with a syncopal episode. The patient reported a history of recurrent pre-syncope and syncope since her teenage years, as well as a family history of sudden cardiac death. Three years prior to her current presentation, the patient had an exercise stress test, 24-hour Holter monitor, and two echocardiograms that were unremarkable. Three weeks prior to presentation, the patient had a cardiac MRI that revealed focal mass hypertrophy of the basal anterior to mid anterior wall measuring up to 2.5 cm. In the ED, the patient was treated with intravenous fluid and beta-blockers; however, beta-blocker therapy had to be discontinued because the patient was experiencing presyncopal episodes and orthostatic hypotension. The patient was started on midodrine with partial improvement lightheadedness, dizziness, and presyncope. The patient was transferred to a tertiary center with the plan to do serial imaging and place an implantable cardioverter-defibrillator (ICD) if the focal mass thickness reached 3 cm and explore surgical intervention if symptoms worsened. Identifying and reporting anomalous variants of HCM is critical for optimal management of patient care and to improve outcomes.

## Introduction

Hypertrophic cardiomyopathy (HCM) is genetic cardiomyopathy, most often caused by an autosomal dominant mutation in cardiac sarcomere proteins which causes ventricular concentric hypertrophy and can lead to sudden cardiac death. The prevalence of HCM is estimated to affect 0.2% of the human population [1]. The clinical presentation of HCM ranges from asymptomatic, to dyspnea on exertion, syncope with exercise, angina, palpitations, and ventricular arrhythmias. The most common phenotypes of HCM manifest mid-ventricular hypertrophy with apical aneurysm, concentric hypertrophy, asymmetric septal hypertrophy, and apical hypertrophy. Although rare, other phenotypic variants of HCM can exist. This case study reports a unique case of mass-like HCM with focal hypertrophy at the basal anterior to mid-anterior left ventricular wall anteroseptal extension with residual contractility. We present a clinically oriented review of HCM spanning disease definition, pathophysiology, family screening, imaging assessment, risk stratification, and therapeutic approaches.

## Case presentation

**Figure 1:**
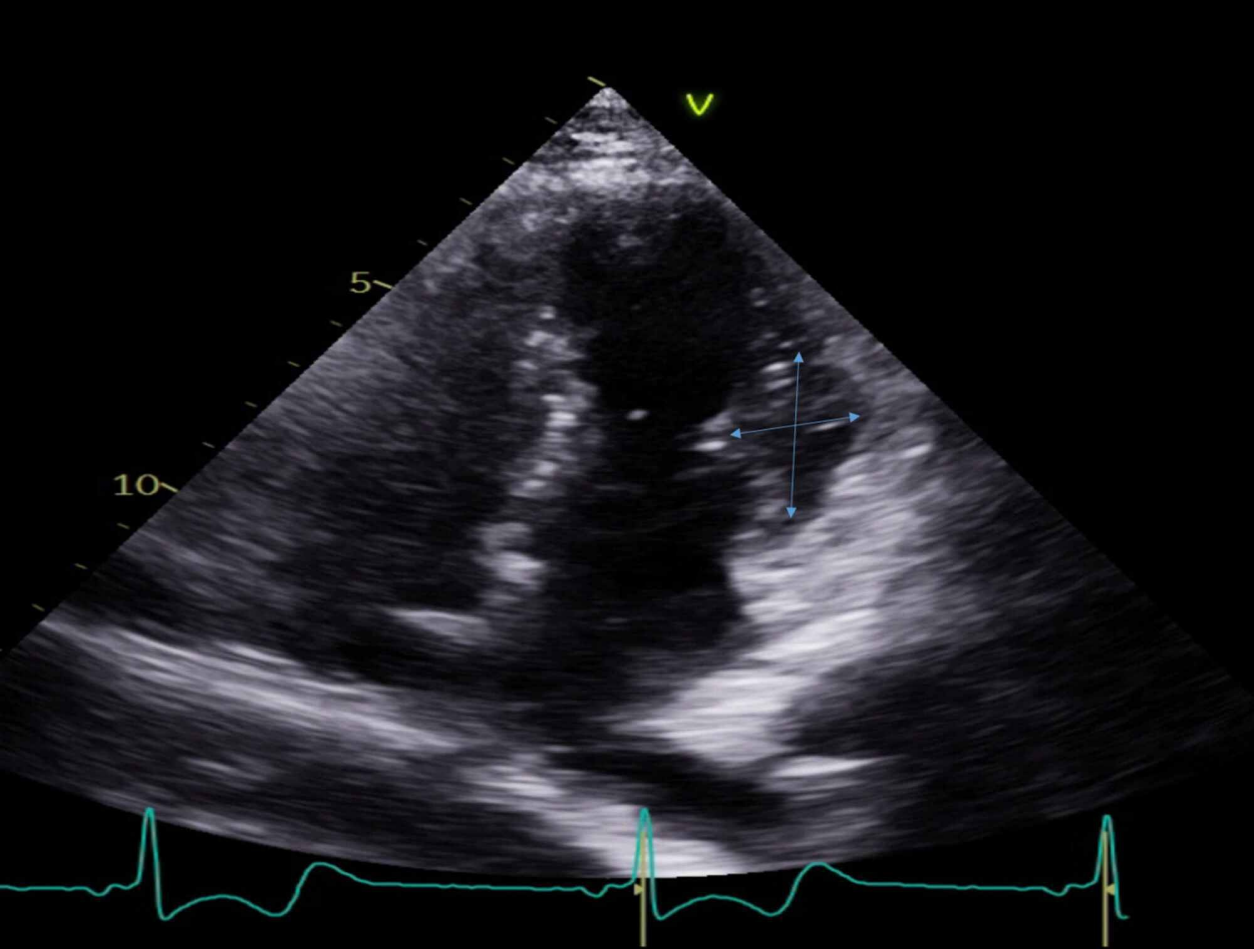
Echocardiogram demonstrating moderate asymmetric septal left ventricular hypertrophy with ejection fraction of 68%, wall motion was within normal limits, peak and mean left ventricular outflow tract (LVOT) gradients less than 4 and 2.5 mm respectively with no increase in gradient with provocative maneuvers such as Valsalva.

**Figure 2:**
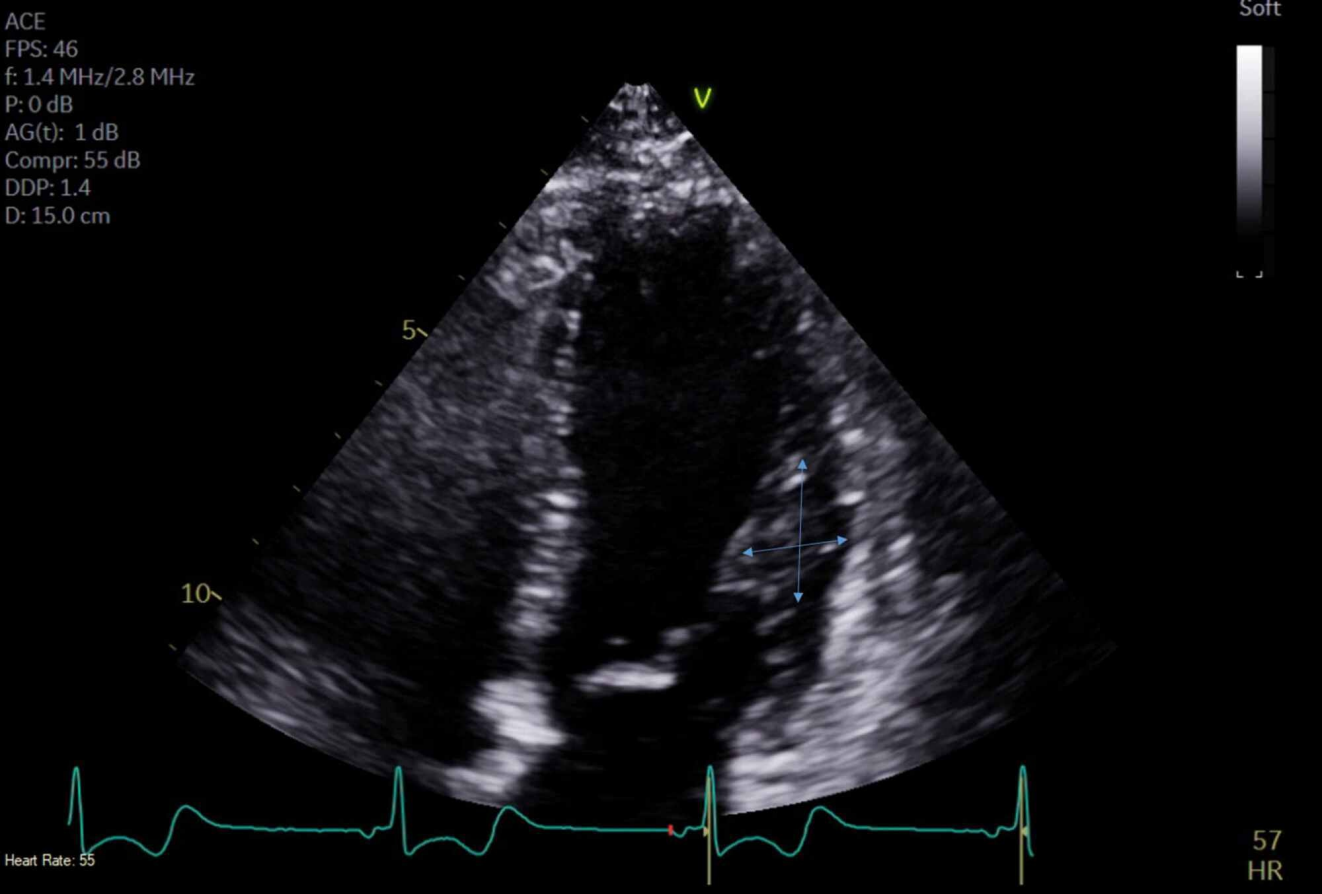
Echocardiogram demonstrating moderate asymmetric septal left ventricular hypertrophy with ejection fraction of 68%, wall motion was within normal limits, peak and mean left ventricular outflow tract (LVOT) gradients less than 4 and 2.5 mm respectively with no increase in gradient with provocative maneuvers such as Valsalva.

**Figure 3:**
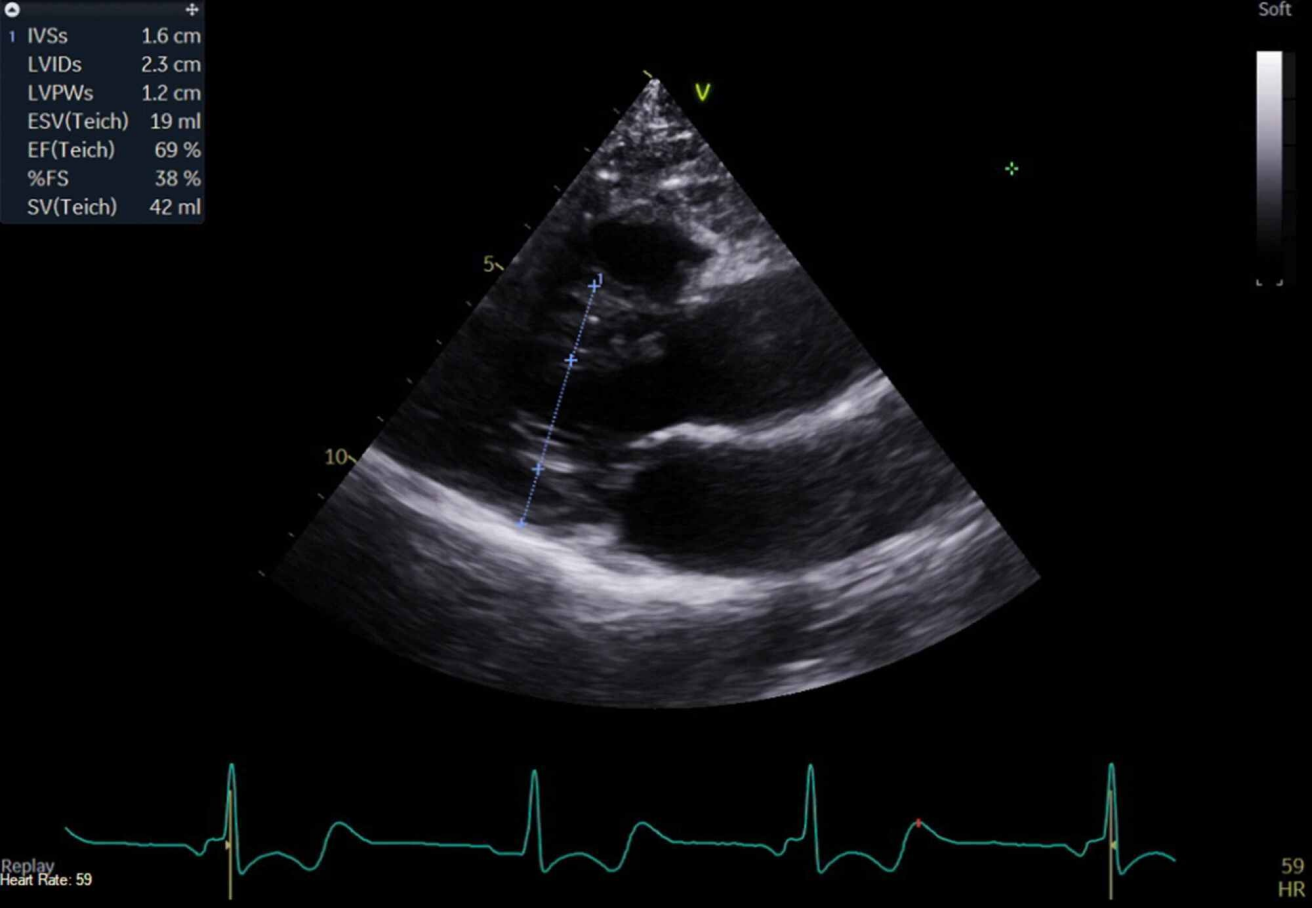
Echocardiogram demonstrating moderate asymmetric septal left ventricular hypertrophy with ejection fraction of 68%, wall motion was within normal limits, peak and mean left ventricular outflow tract (LVOT) gradients less than 4 and 2.5 mm respectively with no increase in gradient with provocative maneuvers such as Valsalva. Intraventricular septum measuring 1.6 cm, but mass located in the basal anterior wall.

A 29-year-old female with a past medical history of recurrent orthostatic hypotension, syncope, anxiety disorder, and depression presented to the emergency department (ED) complaining of lightheadedness, dizziness, and generalized weakness associated with a syncopal episode. On presentation, patient's blood pressure was noted to be 87/54 mmHg with remainder of her vital signs remaining stable. On initial presentation, labs demonstrated normal hemoglobin of 14.5, mean corpuscular volume (MCV) of 92, negative troponin, and electrolytes were within normal limits with sodium of 137, potassium of 3.8, calcium of 9.2, magnesium of 2.0. An electrocardiogram revealed sinus tachycardia, evidence of left ventricular hypertrophy with repolarization abnormality, short PR interval, and nonspecific ST-T wave changes. Echocardiogram revealed moderate asymmetric septal left ventricular hypertrophy with ejection fraction of 68%, wall motion was within normal limits, peak and mean left ventricular outflow tract (LVOT) gradients less than 4 and 2.5 mm respectively with no increase in gradient with provocative maneuvers such as Valsalva (Figures 1, 2, 3).

**Figure 4:**
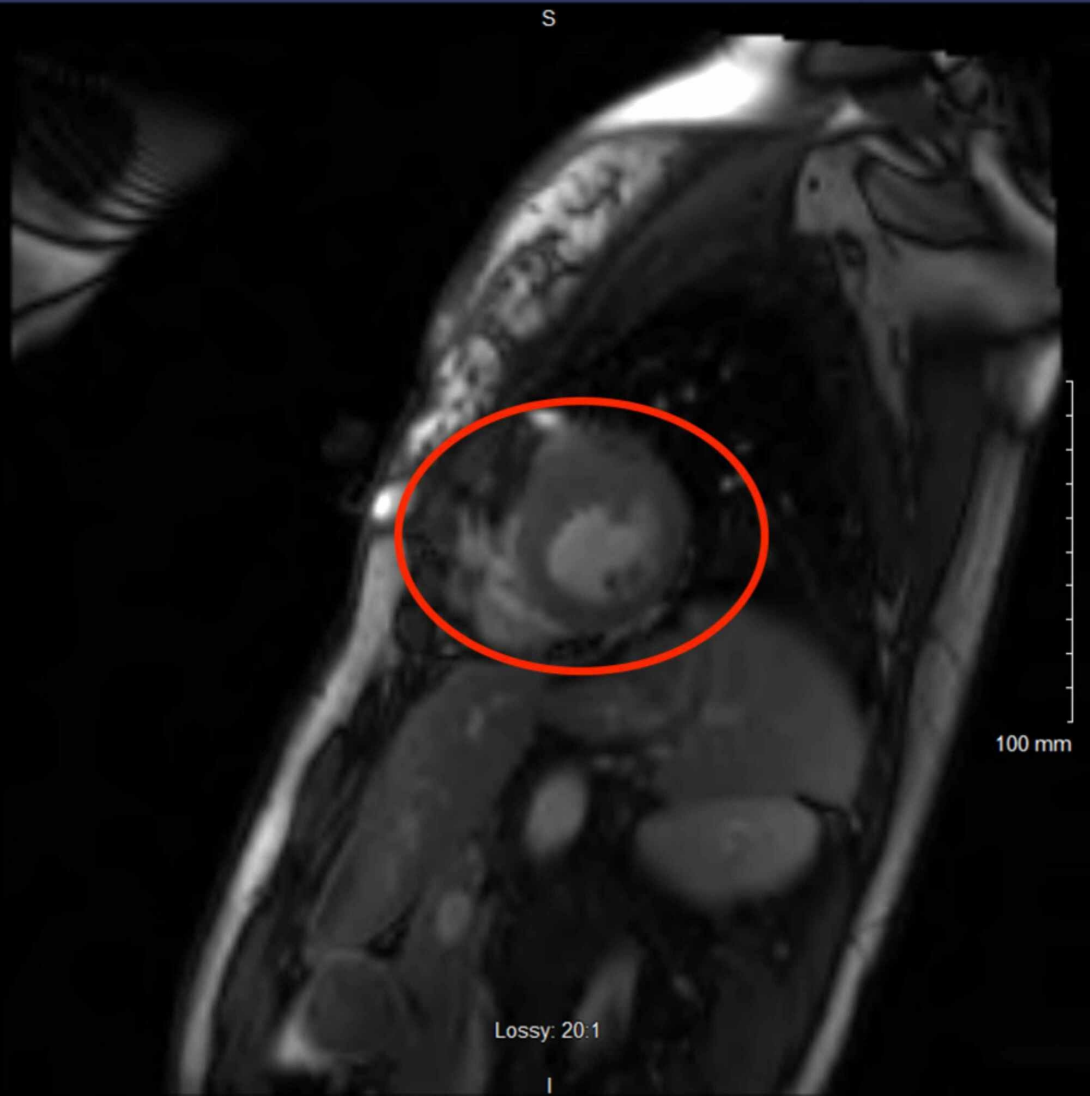
Focal mass hypertrophy of the basal anterior to mid anterior wall measuring up to 2.5 cm along the base with slight extension towards the anteroseptal, remaining of the myocardium was normal in thickness (red oval).

**Figure 5:**
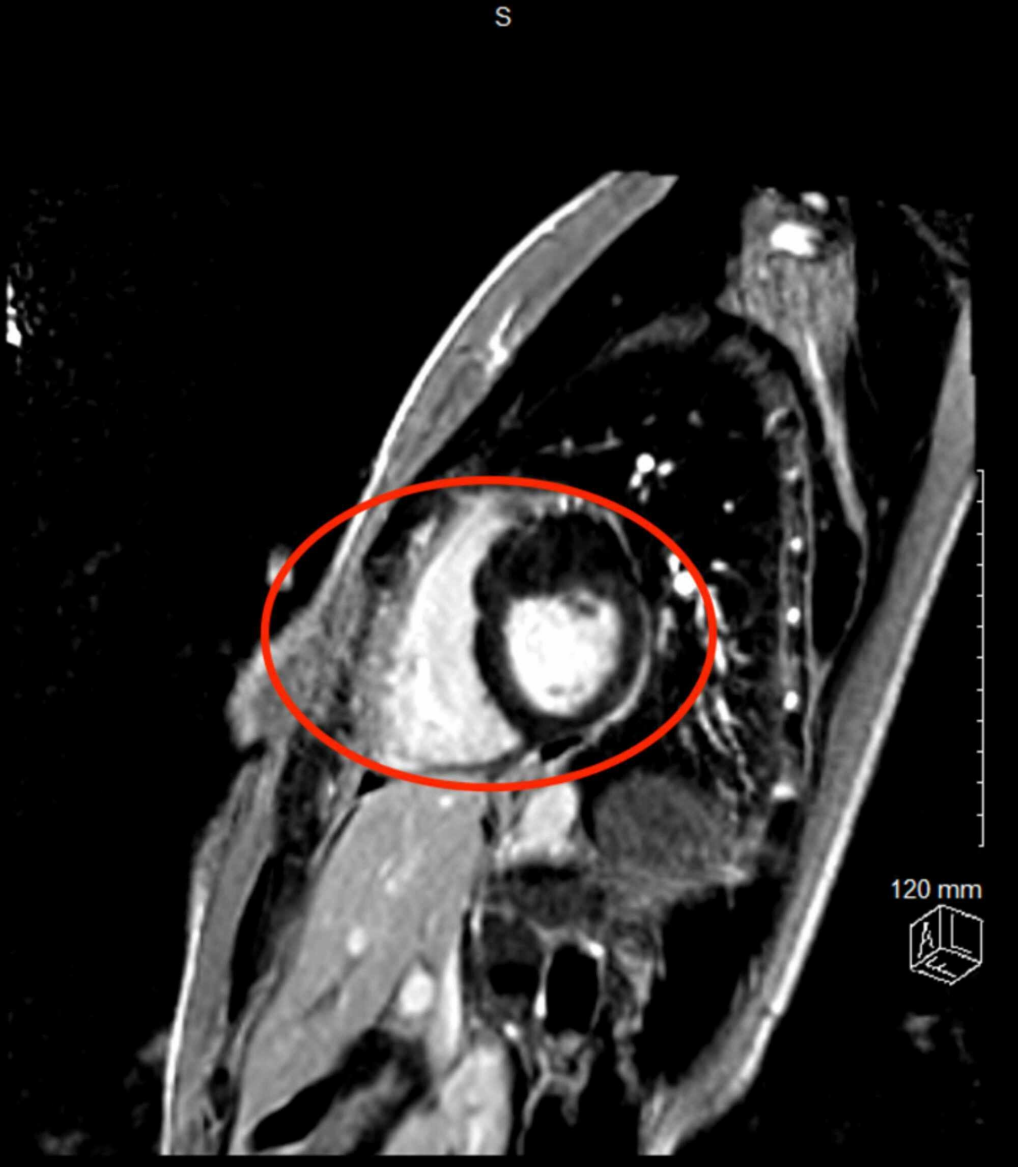
Delayed sequence demonstrating mild patchy, midmyocardial gadolinium enhancement with thickening of anterior wall from the base to mid cavity (red oval).

The patient reported that she had multiple syncopal episodes since her teenage years, a positive family history of sudden cardiac death, and that she had an extensive cardiac workup in the past. The patient’s father died at 54 years old and half-brother at 42 years old; however, autopsy findings of her relatives were not suggestive of structural abnormalities. Furthermore, the patient had a cardiac workup that included two echocardiograms, an exercise tolerance test, a 24-hour Holter monitor, and a cardiac MRI. Her most recent echocardiogram performed one year prior to presentation demonstrated a normal ejection fraction, normal left ventricular size, and normal wall thickness. Her 24-hour Holter monitor performed one year prior to presentation demonstrated normal sinus rhythm with occasional premature ventricular contractions (PVCs), but no evidence of high-grade ectopy or prolonged pauses. An exercise tolerance test performed two years prior to current presentation was unremarkable; of note, the patient did develop a heart rate of 188 bpm and appeared to be in sinus tachycardia but there was no evidence of supraventricular tachycardia, ventricular tachycardia, or definitive ST-segment changes. Cardiac MRI was performed three weeks prior to presentation and showed focal mass hypertrophy of the basal anterior to mid anterior wall measuring up to 2.5 cm along the base with slight extension towards the anteroseptal, remaining of the myocardium was normal in thickness (Figures 4, 5). There was mild subvalvular acceleration of flow likely secondary to some degree of subvalvular stenosis, there was no evidence of systolic anterior motion of the mitral valve during systole, however there was slight anterior motion of the mitral valve seen during late diastolic and thickening of the anterior mitral leaflet was also noted.

During the course of the hospital stay, the patient was treated with intravenous fluid hydration and metoprolol; however, metoprolol was discontinued after the patient experienced symptomatic presyncopal episodes and orthostatic hypotension. The patient continued to have recurrent episodes of presyncope and diaphoresis and was subsequently treated with midodrine to assist with increasing afterload and decreasing gradient, and the patient reported partial improvement lightheadedness, dizziness, and presyncope. She was maintained on cardiac telemetry monitoring throughout her hospital course which did not reveal ventricular tachycardia or supraventricular tachycardia. The cardiac electrophysiologist estimated the patient’s five-year sudden cardiac death risk to be less than 4%, thus an intracardiac device was not indicated. The patient was continued on medical management with hydration, increased salt intake, and avoidance of afterload reducing agents; however, episodes of presyncope persisted.

A multidisciplinary meeting including a radiologist, cardiologist, hospitalist, cardiac electrophysiologist, and cardiothoracic surgeon was held and it was determined that the patient had a rare variant of “mass-like” hypertrophic cardiomyopathy. Eventually, due to refractory symptoms the patient was transferred to a tertiary care center for further management by a cardiomyopathy specialist. and diagnosis of a rare variant of hypertrophic cardiomyopathy was established. The plan was to hold beta-blockers as patient could not tolerate and perform serial imaging with plans for implantable cardioverter-defibrillator (ICD) placement if the focal mass thickness reached 3 cm and surgical intervention if the symptoms worsened.

## Discussion

HCM is one of the most common primary causes of heritable cardiomyopathy, and degree of left ventricular hypertrophy and left ventricular configuration varies based on phenotype [2]. Patients present with symptoms ranging from asymptomatic to decompensated heart failure and even to sudden cardiac death. This variable presentation occurs from abnormal left ventricular configuration and varied dynamic left ventricular outflow obstruction [2]. These patients may eventually develop progressive left ventricular hypertrophy as a consequence of hemodynamic circulation [3]. Hence, the therapeutic goal targets to decrease the dynamic obstruction and improve hemodynamic circulatory parameters [1]. It is estimated that HCM may be affecting one in 500 people and some recent investigations have suggested higher prevalence [4]. About 70% of HCM patients have left ventricular outflow tract obstruction defined as left ventricular outflow tract gradient of more than 30 mmHg either with provocation or even occurring at rest [5]. A lesser percentage of patients who do not typically present with the features of left ventricular outflow tract obstruction; however, it is noted that LVOT can vary with fluctuation in the fluid status of the body; factors including autonomic activity, exercise, medications also play a role in symptom presentation. Typically, septal hypertrophy or abnormal subvalvular mitral apparatus can result in turbulent blood flow resulting in drag force causing systolic anterior motion of the mitral valve and decreased forward flow [5]. This hemodynamic compromise can result in presyncope as well as syncope in up to one in four patients with HCM. There are associated cardiac arrhythmias including sinus node dysfunction, complete heart block, ventricular arrhythmias, and supraventricular arrhythmias that can all result in low cardiac output symptoms [6]. 

**Figure 6:**
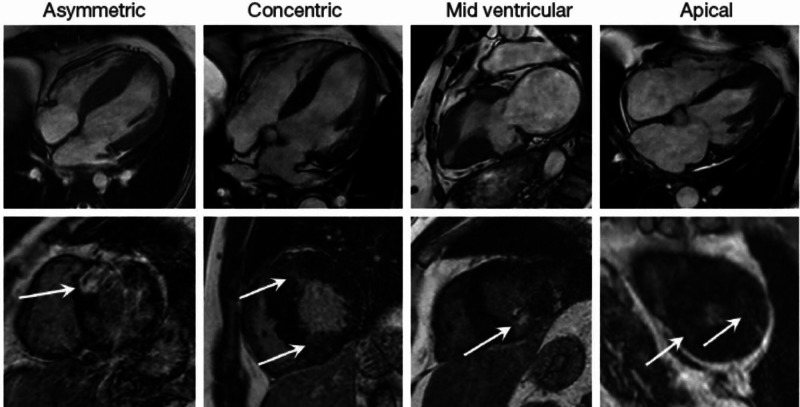
Selection of the most common HCM phenotypes assessed with MRI. Diastolic SSFP images (top row) with corresponding late gadolinium enhancement images (bottom row) illustrating, asymmetric septal hypertrophy, concentric hypertrophy, mid-ventricular hypertrophy with an apical aneurysm and apical hypertrophy. In all cases there is patchy mid-wall fibrosis demonstrated on the late gadolinium enhanced images (arrows). HCM, hypertrophic cardiomyopathy; MRI, magnetic resonance imaging; SSFP, steady state free precession

As the patient presentation is phenotype-dependent and based on the disease severity and underlying defect affecting different structural and functional proteins of the cardiomyocyte, sarcomere hypertrophic cardiomyopathy is more frequently seen, however metabolic diseases including glycogen storage diseases, extracellular deposition diseases like amyloidosis, and mitochondrial diseases may all resolved and myocardial hypertrophy [3]. Hence, family screening strategies and risk assessment are as important as specific therapies for patients with HCM. Most common phenotypes of HCM as assessed with MRI are mid-ventricular hypertrophy with apical aneurysm, concentric hypertrophy, asymmetric septal hypertrophy, and apical hypertrophy, however patient had focal hypertrophy of the basal anterior to mid anterior left ventricular wall of 2.5 cm along the base, with slight extension towards the anteroseptal (Figures 4, 5). This focal hypertrophy, also known as masslike HCM, can mimic the appearance of myocardial based tumor-like fibroma with the key discriminatory feature on MRI for residual contractility with HCM as compared to fibroma, which shows uniform and intense late gadolinium enhancement as compared with HCM [7]. Our patient was symptomatic with recurrent episodes of presyncope and syncope despite no evidence of left ventricular outflow tract obstruction or any significant cardiac arrhythmias noted on telemetry can be explained by the labile nature of the outflow obstruction. “Mass-like” HCM can mimic the appearance of a myocardial based tumor such as a fibroma; the key discriminating features on MRI are the presence of residual contractility with HCM whereas a fibroma displays intense and uniform late gadolinium-enhanced (LGE) (Figure 6) [7]. Right ventricular hypertrophy has been reported in 15-20% of HCM patients, and most often involves the mid-to-apical portion of the right ventricle, often contiguous with left ventricular hypertrophy (LVH). There are sporadic case reports of HCM causing right ventricular outflow tract obstruction [8].

Management of these patients starts by reevaluating patient's pharmacotherapy that can worsen dynamic left ventricular outflow tract obstruction which includes diuretics, vasodilators, and beta agonists. Beta-blockers are the mainline therapy that cause negative inotropy as well as negative chronotropy, which reduces dynamic left ventricular outflow tract obstruction and prolongs diastole improving left ventricular filling, respectively [9]. Alternatively, non-dihydropyridine calcium receptor blockers can also be used if beta-blockers are not tolerated due to side effects. Other medications used are disopyramide, which is a class I antiarrhythmic agent with negative inotropic effects however mostly combined in conjunction with other therapies. Perhexiline, which is a potent inhibitor of carnitine palmitoyltransferase-1 and possesses anti-inflammatory properties, has also been poorly defined for use in HCM patients. Aldosterone receptor blockers to help reduce left ventricular hypertrophy are also being investigated [10]. However, the patient in this case was unable to tolerate beta-blockers due to borderline low blood pressure and hence was maintained on nonpharmacological options including adequate oral hydration, adequate salt intake to avoid hypotension, and avoidance of strenuous activities. 

Dynamic LVOT obstruction, defined as LVOT gradient ≥30 mmHg, is a determinant of a therapeutic approach to HCM [5]. Approximately 70% of HCM patients have LVOT obstruction at rest or with provocation [5]. Physiologically, narrowing of the LVOT secondary to septal hypertrophy creates turbulence and causes decreased forward flow and systolic anterior motion (SAM) that can subsequently cause syncope. LVOT in HCM is labile and is influenced by volume status, autonomic stimulation, pharmacotherapy, exercise, etc [6]. Syncope and pre-syncope occur in one in four HCM patients and is secondary to numerous mechanisms ranging from arrhythmias to LVOT to hypotension. Unexplained, non-neurocardiogenic syncope, particularly if recent (<six months), corresponds to an increased risk of sudden cardiac death (SCD) [11]. Both American College of Cardiology Foundation (ACCF)/American Heart Association (AHA) guidelines (Class IIa) and European Society of Cardiology (ESC) guidelines include the unexplained syncope in ICD decision making. LVOT obstruction has been linked to SCD and is a component of the HCM Risk-SCD Calculator [11].

An HCM Risk-SCD Calculator has been developed to determine the risk of sudden cardiac death and assist with ICD decision-making. The incidence of SCD in patients with HCM is 0.7% per year; however, it increases to 3-5% per year in certain subgroups of patients [7]. Thus, it is crucial to identify high-risk patients who will benefit from prophylactic ICD therapy. Conventional risk stratification model uses five clinical markers for primary prevention (applicable to HCM patients without prior cardiac arrest) that include: premature SCD (presumably caused by HCM) in one or more relatives, unexplained syncope, non-sustained ventricular tachycardia (VT) on ambulatory Holter monitoring (particularly when multiple, repetitive or prolonged), severe LV hypertrophy (maximal wall thickness >30mm), and abnormal blood pressure response during exercise [9]. It is to be noted that the absence of all risk factors does not confer immunity to SCD. Age is an important factor while considering these individual risks and is greatest when present among individuals less than 50 years. The SCD risk increases according to the number of risk factors and incorporation of “non-conventional” potential risk modifiers (end-stage HCM with left ventricular ejection fraction (LVEF) <50%, LV apical aneurysm, extensive LGE on cardiac MRI, significant LV outflow tract gradient at rest, multiple sarcomere mutations, previous alcohol septal ablation and other modifiable factors such as intensive exercise, CAD) [9]. The ESC has incorporated a novel approach to risk stratification in their 2014 guidelines. While the conventional model described above is based on the sum of the established risk factors, the “HCM risk-SCD” model (http://www.doc2do.com/hcm/webHCM.html) calculates an individual’s five-year risk of SCD. Seven continuous or binary risk factors are used to stratify patients into one of the three groups; if the calculated five-year risk is <4%, an ICD would not be warranted; if the calculated risk is between 4-6%, it would be contingent and if above 6%, an ICD implantation would be warranted [12].

From a pharmacologic perspective, the primary goal of HCM management is symptom reduction and treating comorbidities independent of HCM [9]. First, it is important to do a medication reconciliation and review if the patient is taking medications that can worsen LVOT including digitalis, vasodilators, and diuretics. Next, it is important to introduce pharmacotherapy that can improve symptoms such as beta-receptor antagonists, nondihydropyridine calcium receptor antagonists, Class Ia antiarrhythmics, and other novel drugs. Beta-receptor antagonists are the mainstay of therapy because their negative inotropic and negative chronotropic effects that synergistically act to reduce dynamic LVOT obstruction, prolong diastole, blunt adrenergic mediated tachycardia, and minimize ischemic supply-demand mismatch [9]. Nondihydropyridine calcium receptor antagonists are an alternative to beta-receptor antagonists if there are unacceptable side effects or inadequate symptom relief with beta-blockers. Concomitant calcium and beta-receptor antagonists may be administered, but can increase the risk of hypotension, atrioventricular block, and sinus node dysfunction. Class Ia antiarrhythmics with negative inotropic effects, such as disopyramide, can be used alone or in conjunction with other therapies; however, they should be used with caution as they are associated with proarrhythmic effects, significant anticholinergic side effects, and are costly for patients. Other therapeutic agents for HCM are being explored, such as perhexiline (a weak calcium channel antagonist with potent inhibition of carnitine palmitoyltransferase-1 and anti-inflammatory properties), and aldosterone receptor blockers, although no clinical role is yet established in HCM [10].

## Conclusions

HCM is a common form of cardiomyopathy that can have life-threatening complications. As more data comes out suggesting an even higher prevalence in HCM across the world, it is important to understand the pathophysiology of the disease and its phenotypic variations, such as mass-like HCM. In this case, the patient had an extensive cardiac workup in the past that was clinically suggestive of HCM but diagnostically unremarkable. Hence, the diagnostic utility of cardiac MRI can play a critical role in the diagnosis of HCM, and can aid in the differentiation between cardiac tumors and HCM. Establishing the correct diagnosis is critical to tailor optimal medical therapy to improve quality of life for HCM patients.
